# Accepting the challenge: what academic health sciences library directors do to become effective leaders

**DOI:** 10.5195/jmla.2018.350

**Published:** 2018-04-01

**Authors:** Rick L. Fought, Mitsunori Misawa

**Affiliations:** Associate Professor and Director, Health Sciences Library, University of Tennessee Health Science Center, Memphis, TN; Assistant Professor, Adult Learning (PhD) and Adult Education (MS), University of Tennessee, Knoxville, TN

## Abstract

**Objective:**

This study sought to better understand effective leadership through the lived experiences of academic health sciences library directors.

**Methods:**

Phenomenological interviews were conducted with eight academic health sciences library directors to capture the essence of their shared leadership experiences. The research question that guided the study was: How do academic health sciences library directors understand their leadership effectiveness? The interviews were transcribed and coded, and the data were analyzed thematically.

**Results:**

Three main themes emerged from data after analysis: assessment of the environment, strategies and decisions, and critical skills. Assessment of the environment includes awareness not only of trends in libraries and technology, but also the trends in health information, higher education, and current events and politics of their institutions and states. The strategies and decisions theme is about the ability to think both in the long-term and short-term when leading the library. Finally, critical skills are those leadership skills that the research participants identified as most important to their leadership effectiveness.

**Conclusions:**

The study identified three main themes capturing the essence of the research participants’ leadership experiences. The three themes constitute a wide array of leadership skills that are important to learn, understand, and develop to increase leadership effectiveness. Effective leadership is fundamental to obtaining long-term strategic goals and is critical to the long-term future of the libraries.

## INTRODUCTION

Effective leadership can be defined as the successful application of influence toward goal completion [1]. Rosser et al. describe effective leaders as “able to obtain the cooperation of other people and to harness the resources provided by that cooperation to attain organizational goals” [2]. It can be difficult to define and explain leadership effectiveness in higher education: “Higher education’s distinctive combination of goals, tasks, employees, governance structures, values, technologies, and history makes it not quite like anything else” [3]. Perhaps as a result, there is a paucity of research directly addressing the daily activities and overall effectiveness of academic deans [4].

The same is true in regard to studies of leadership effectiveness in academic libraries. Fagan commented that the studies that do exist appear to have been done in relative isolation from one another [5]. The studies have also tended to be concentrated in only a few areas of leadership effectiveness theory. Only one leadership study, conducted by the authors of this paper, has specifically addressed the effectiveness of library directors in academic health sciences libraries in the United States [6]. This is unfortunate because a number of studies have discussed how effective leadership is central to the success and future of academic libraries [6–9]. Fair or not, the perceived effectiveness of the library director and the library are closely associated, and adequate funding for the library, a key indicator of administrative support, is primarily determined by the administration’s confidence in the leadership of the library [10].

There has been little agreement on even what constitutes effective leadership in higher education [2, 11]. In fact, Fincher contends that leadership effectiveness in higher education is mostly based on perceptions and what leaders are perceived to have accomplished [12]. The study concludes that leaders are defined in terms of their abilities and the activities required to fulfill institutional functions and purposes [12].

Rosser et al. attempted to present “a systematic approach for evaluating the leadership effectiveness of deans and directors from individual and institutional perspectives.” They noted that academic deans are most often judged effective or ineffective via informal assessments. They developed a model that takes into account the relevant dimensions of leadership and the multilevel nature of higher education institutions [2]. Effective leadership in academic libraries is even less understood, with research being scattered and few connections existing between studies not written by the same authors [5]. Academic health sciences libraries are similar to other academic libraries but do face unique challenges that their directors must handle to be effective leaders [6].

Therefore, the purpose of this study was to better clarify the understanding of effective leadership in academic health sciences libraries through an examination of library directors’ leadership experiences. The research question that guided the study centered on capturing how directors at these libraries understood effective leadership through an examination of their shared experience as library leaders and how they defined and explained the effectiveness of their leadership. The research question that guided the study was: How do academic health sciences library directors understand their leadership effectiveness?

## METHODS

The research design for this study followed phenomenology theory, which focuses on revealing meaning and understanding the essence of a shared experience and works very well with phenomena that do not lend themselves to easy quantification, like leadership [13]. Phenomenology searches for the essence of a phenomenon from people’s shared experience of it, which cannot be revealed by ordinary observation, and analyzes this phenomenon for themes and meanings so it can be described and understood [14]. Phenomenological research was deemed the most suitable methodology for comprehending how academic health sciences library directors developed their understanding of effective leadership due to its usefulness in understanding subjective experience and gaining insight into an individual’s motivations and actions [15, 16].

Rosser et al.’s model of seven domains of leadership responsibility served as the theoretical framework to guide the analysis and discussion of the study. The model’s seven domains represent central evaluation criteria regarding the responsibilities and skills necessary for an academic dean and translate well for evaluating the effectiveness of academic library directors since their institutional roles are quite similar. Rosser et al. noted that academic deanships were leadership roles that were much more political and social than hierarchical and technical, which would also seem to describe library director roles [2]. The model provides a comprehensive and practical approach to interpreting and understanding the research findings.

The study used purposeful sampling with criterion-based sampling strategies to select its research participants. Purposeful sampling selects information-rich cases or participants to study, which by their nature and substance will explain and clarify the research questions [17]. Criterion-based sampling reviews and studies all cases or participants that meet a predetermined criterion of importance and eliminates any cases or participants that do not meet the predetermined criterion [17].

Participants for the study met the following predetermined criterion: they were academic health sciences library directors at public research universities with “very high research activity” in the Carnegie Classification of Institutions of Higher Education. The research participants were further limited by geographic location, with the focus being primarily on institutions in the Southern region of the United States. The final criterion was active membership in the Association of Academic Health Sciences Libraries. The intention behind these selection criteria was to identify library directors at larger institutions who would hopefully have a larger range of experience and would be geographically closer to the researchers to allow the possibility of on-site interviews. Thirteen academic health sciences library directors met the criteria and were invited to participate, with eight agreeing to join the study. Approval from the governing institutional review board was granted prior to contact with the research participants for this study.

In-depth, phenomenologically based, semi-structured interviews are the primary method of data collection in phenomenological research, wherein the researcher attempts to uncover the essence of the meaning of the experience [18]. Phenomenological-based interviewing, therefore, focuses on the experiences of the participants and the meaning they make from those experiences [18]. The interviews with the research participants of this study were conducted over the phone and recorded to ensure completeness and accuracy. The interviews were later transcribed.

Interview data were analyzed using thematic analysis, which is a general approach that prepares and organizes data for analysis by first coding the data, then analyzing the codes for patterns that can be organized into categories, and finally analyzing these categories for emergent themes [19, 20]. Qualitative codes capture the essence and essential elements of the research story. When clustered together, because of similarity and regularity, patterns emerge that facilitate the development of categories, which are then used to develop broader themes [20].

This study relied on member checking, peer debriefing, and thorough and rigorous designing of the research to ensure trustworthiness. Member checking allows the research participants the opportunity to clarify their answers and comment as to whether the interview fully reflected their experience of the phenomenon that was discussed [21]. Participants may also be asked to later comment on the credibility and accuracy of the findings and interpretations from the study. Peer debriefing is an external check of the research process and provides an independent viewpoint for the researcher. The peer debriefer keeps the researchers honest and asks hard questions about the methods and interpretations of the study to ensure reliability and trustworthiness [21].

Subjectivity or bias is present throughout the research process. It is, therefore, important for researchers to systematically identify their subjectivities, assumptions, and stereotypes throughout the course of their research [22, 23]. Addressing the issue of subjectivity does not eliminate the subjectivities; however, it does enable the researchers to manage them, minimize their impact on the research process, and achieve a better understanding of themselves [22, 23]. In this study, one researcher is an experienced librarian in a leadership position at an academic health sciences library, and the other is a professor of education with no prior work in libraries. Both are very interested in the study of leadership effectiveness in higher education.

## RESULTS

After transcribing and coding the interviews of the eight research participants and then performing thematic analysis, three themes emerged that were relevant to effective leadership in academic health sciences libraries: assessment of the environment, strategies and decisions, and critical skills. Pseudonyms have been used to protect the identities of the research participants.

### Assessment of the environment

The large majority of the interviewed research participants spoke of the need for academic health sciences library directors to be aware of not only trends in libraries and technology, but also the trends in health information, higher education, and the current events and politics of their institutions and states. It is from this information gathering and awareness of current trends that directors are able to understand the context of their libraries in their institutions and then make informed decisions and plans for their libraries. Gretchen, a library director for over fifteen years, described this in her comments on effective library directors: “They look at the trends in their environment. They look at their environment at an institutional level. [They] need to be able to know how to navigate the politics and the political landscape of their institution.” Gretchen elaborated on this point: “[T]he most important thing is being able to stay on top of [trends and your environment] and be nimble. You have to put yourself in a place where you know what’s happening and be able to react appropriately.”

Other research participants expressed similar sentiments. Maria, who has been a library director for over ten years, said that effective library leaders are able to “look at a situation and grasp the essence of it in what [they] actually need to do, and don’t get sidetracked.” In other words, they understand their environment, what their role is, and what they need to do to be effective. Janet, a director with more than thirty years of experience, described her approach to challenges and her efforts to be effective as, first, “assess[ing] the status of where [her] library is, identifying where it needs to go, and then realign[ing] resources and energy to take in that direction.”

The general consensus from the research participants, in their shared experience as academic health sciences library directors, was that assessing their environments and staying current with trends and their institutions was a critical part of their job if they wanted to be effective. They considered it necessary to be able to find and take advantage of opportunities as well as most effectively managing the challenges they faced.

### Strategies and decisions

The next theme addresses the long-term and short-term planning skills that academic health sciences library directors need to develop to increase their effectiveness. Directors need to balance the decisions that they are required to make daily with the overall strategy and goals that they are striving to achieve. It is a balance of operational effectiveness joined with long-term strategic planning to achieve the goals of the library.

Janet discussed this when asked to describe effective leadership in academic health sciences libraries. She spoke about the director having “to know where the library needs to be in a couple of years, in five years, and in ten years.” Library directors “need to know how they are going to get [their libraries] to the next stage” and “be willing to make some tough choices.” Janet explained that making the tough decisions would mean upsetting some people, but “at the end of the day, you need to believe that you know that you did, not what was good for you, but what was good for the organization.” Janet’s thoughts on the matter are perhaps best summarized as:

My responsibility is the future of the library, and I’m going to be doing what I believe is in [its] best interest. Sometimes that’s going to mean making a tough decision that isn’t necessarily always the most popular one.

These same sentiments were expressed by a majority of the research participants. John, who was serving as interim director of his library and had many years of experience working in academic health sciences libraries, stated that he believed making decisions was a principal responsibility of a leader and that it was an important responsibility:

Ultimately, the responsibility [for making decisions] rests with [the director]. It is damaging to an organization if it is not clear that somebody is going to make the decision, tell everyone what it is, be able to stand by [the decision].

John also thought this was an important contribution to library directors being respected and trusted by their staff.

Strategic planning and visioning are long-term planning skills necessary for library directors to be effective. These skills enable directors to chart the future course of their libraries, and every research participant spoke about the importance of getting this aspect of leadership right. Lisa, who had been a library director for more than fifteen years, said she liked to fully think through where she wanted her library to move because “nothing will lose your troops faster than changing directions quickly and often.” Effective leadership is ultimately about achieving goals and objectives [1, 2]; therefore, planning and choosing those goals are of paramount importance.

Peggy, a library director for over ten years, had this to say about what strategic planning meant to her:

[M]y judge is looking at our strategic plan. [Is] it what we said we were going to do. Now, did we achieve it? It lets me know if I have been effective and whether I have given my people what they need to do their jobs. Have I given them the resources, people, and money they need. If we are achieving the goals that we set in our strategic plan, then I feel like I have been effective.

Long-term strategic planning and visioning can take up time and resources, but it forms the foundation for library directors to be effective leaders. Donna, another library director for over ten years, described how “our strategic planning endeavor took a lot of time, and it should take a lot of time because it is important and you need to get it right.”

### Critical skills

The research participants noted a number of skills that they considered critical to effectively leading their libraries. A number of these were discussed in a previous article by the authors, such as developing key relationships and advocating for the library [6]. Other skills were also mentioned that research participants believed were essential to their effectiveness as library directors.

First, good leaders know how to build a team and recruit the best people to join them at their library. Lisa advised, “you need a really strong grasp of human resources and I mean in interviewing, picking the right people, and being able to identify the qualities in the people that you want on your team.” Directors must have good teams to work with if they want to accomplish the goals of their libraries, take advantage of opportunities, and effectively run their libraries. As Peggy explained, “I am more like a guide. I hire terrific people to operate the library and they are my most valuable resource. They are the ones helping students with projects or helping researchers.” Donna likened the library director role to that of being an orchestral leader: “you want everybody playing at their peak and playing to create good things, so hire good people, give them direction and counsel, and then let them work.” As a final point on the importance of building a team and hiring good people, Janet advised, “people that are good leaders are not threatened by really smart, talented people. I’ve seen a lot of people that were, and a good leader is happy to have somebody smart working for them.”

Next, effective library directors understand how to develop their staff and encourage people to grow professionally to improve their performance. Maria, for example, spoke about helping her staff to overcome hurdles and how library directors needed to “clear the way and allow people to grow.” Her sentiments were closely matched with what Janet said on the topic:

I have a lot of conversations with my people about what it is we’re trying to do, and why we are doing this, and what does success look like here? But I don’t tell them necessarily how to do it. We talk about it in a broad sense, but they know better than I do and I need to get out of their way.

Finally, effective library leaders know how to communicate and persuade on behalf of the library. Bob, who has been an academic health sciences library director for over twenty years, described how the functioning of his library was often based on his relationships with his supervisors and his ability to communicate his library’s value and its needs. Gretchen stated that her most important role as director was:

to make sure that the library is in the conversation on campus when issues are being discussed and initiatives are being planned. I think one of the most important things I can do is to make sure the library is at the table and has a presence, and is participating in those discussions.

Gretchen also spoke about how important it was that the library director be able to persuade and garner support for the library when necessary: “it’s really being able to make sure that you make the case and that administrators understand what you need and what is going to happen if they do not give you what you asked for.” Donna concurred and said, “I think that probably one of the most important things that a director can have is strong communication skills, including talking to people, hearing what they actually say, and being able to write persuasively to make a case for what is needed for your library.” This was consistently highlighted by each of the research participants as a critical skill for effective library leadership.

## DISCUSSION

This study sought to better clarify understanding of effective leadership in academic health sciences libraries through an examination of library directors’ leadership experiences and to determine the essence of their shared experience as library directors. The findings of the study were generally consistent with other studies examining leadership effectiveness in academic libraries but increase understanding of effective leadership in a couple of notable ways. For example, Young et al. conducted a delphi study with ten Generation X librarians to determine what attributes they perceived as important for academic library leaders to possess to be effective [24]. The authors discovered that the majority of the attributes identified were related to communication and interpersonal skills and concluded that Generation X librarians appeared to prefer participative or supportive leadership styles for creating a more nurturing working environment [24].

Lynch et al., on the other hand, used a different approach and studied the attitudes of university presidents and provosts toward their libraries, using structured interviews. Two of their interview questions addressed the presidents’ views of their library directors’ effectiveness, and several respondents referred to their library directors’ proactive participation in university affairs, helping to forward the universities’ agendas and providing the presidents or provosts with important information for governance and planning. Lynch et al. recommended that library directors learn to operate as team members of the president or provost’s council, delegate more authority to library associate deans, and make explicit connections between the library and the university mission [8]. Both of these studies’ findings were present in this current study in regard to communication and interpersonal skills and in engagement in campus activities and politics. However, the findings in the assessment of the environment theme expanded further on campus dynamics and demonstrate the importance of library directors fully understanding their situations and contexts from a campus, local, state, and national perspective. The authors also found that in addition to good communication and interpersonal skills in general, the interviewed library directors emphasized their need to be able to persuade and advocate to be effective leaders.

Rosser et al. developed a model of seven domains of leadership responsibility to assist in assessing the leadership effectiveness of academic deans and directors. The first two domains of their model were vision and goal setting and management of the unit, and fit our findings in the strategies and decisions theme very well. All of the research participants understood that these were critical skills to their effectiveness as leaders. It was clear that they dedicated a significant amount of time, energy, and resources toward visioning and strategic planning. As Bob put it, “the reality is, unless we have vision and develop goals to guide us, we are rudderless, we don’t have a direction, and we flounder.”

The management of the unit domain is related to the operational effectiveness of the library. Again, this was present in the strategies and decisions theme, which highlighted the importance of library directors making decisions and managing library operations. Whereas vision and goal setting are more long-term planning skills, managing the library and making decisions are more short-term in nature and pertain to the day-to-day operational effectiveness of the library. John, for example, mentioned the administrative duties inherent in a director position, such as delegating work effectively, allocating resources and managing the budget, creating and following administrative policies, and managing staff and change well. The findings highlight how important both long-term strategic planning and day-to-day operational and administrative decisions are for academic health sciences library directors to be effective and to accomplish their goals. They also further understanding of effective leadership in academic health sciences libraries.

The implications of this study are far reaching. Libraries are under increasing pressure to document and articulate their value and the contributions they make to the institutional mission and goals [9]. Higher education in general is under greater scrutiny, and government interest in the effectiveness and accountability of higher education continues to increase [25, 26]. In these uncertain times when libraries are faced with a number of challenges, it is important to have effective leadership for libraries to maintain their prominent educational role on campuses. As several studies have shown, an effective library contributes significantly to improvements in student retention, student success, graduation rates, and grant funding [9, 27–31].

As stated earlier, the perceived effectiveness of the library director and of the library are closely associated and adequate funding for the library, a key indicator of administrative support, is primarily determined by the administration’s confidence in the leadership of the library [10]. Hernon argues that with all the challenges and uncertainty that libraries face, effective leadership is key to their future [32]. The findings of this study help further understanding of effective leadership in academic health sciences libraries and how to improve upon this effectiveness.

Limitations of this study imply possible directions for future research on leadership effectiveness in academic health sciences libraries. The study was limited geographically to libraries in the Southern region of the United States. This was done to provide the researchers the option of doing in-person interviews with the research participants and possibly even some nonparticipant observation. A larger scale study without the geographic limitations is recommended for the future. Also, only one interview was conducted with each participant that lasted between forty-five and sixty minutes. It might have been beneficial to either extend this time or conduct a second interview to get a richer and fuller understanding of the participants’ experiences. Finally, actually being able to visit each participant’s library and conduct the interviews in-person would have provided more context and evidence of their effectiveness as library leaders.

This study sought to determine the essence of research participants’ shared experiences as academic health sciences library directors as they pertain to leadership effectiveness. The study was able to identify three main themes that capture the essence of the leadership experience of the research participants. This identification guided and furthered our understanding of effective library leadership, which is critical in the challenging times that libraries face today. The three themes—assessment of the environment, strategies and decisions, and critical skills—constitute a wide array of leadership skills that are important to learn, understand, and develop to increase leadership effectiveness and improve the likelihood of obtaining the library’s long-term strategic goals.

## References

[1] Chemers MM, Chemmers MM, Ayman R (1993). An integrative theory of leadership. Leadership theory and research: perspectives and directions.

[2] Rosser VJ, Johnsrud LK, Heck RH (2003). Academic deans and directors: assessing their effectiveness from individual and institutional perspectives. J High Educ.

[3] Bolman LG, Gallos JV (2011). Reframing academic leadership.

[4] Wolverton M, Gmelch WH, Montez J, Nies CT (2001). The changing nature of the academic deanship. ASHE-ERIC High Educ Rep.

[5] Fagan JC (2012). The effectiveness of academic library deans and directors. Libr Leadersh Manag.

[6] Fought RL, Misawa M (2016). Effective leadership in academic health sciences libraries: a qualitative phenomenological study. J Libr Adm.

[7] Giesecke J (2010). Finding the right metaphor: restructuring, realigning, and repackaging today’s research libraries. J Libr Adm.

[8] Lynch BP, Murray-Rust C, Parker SE, Turner D, Walker DP, Wilkinson FC, Zimmerman J (2007). Attitudes of presidents and provosts on the university library. Coll Res Libr.

[9] Oakleaf M (2010). Value of academic libraries: a comprehensive research review and report.

[10] Weiner SG (2003). Leadership of academic libraries: a literature review. Educ Libr.

[11] Del Favero M (2005). The social dimension of academic discipline as a discriminator of academic deans’ administrative behaviors. Rev High Educ.

[12] Fincher C, Smart JC (1996). Theory and research in administrative leadership. Higher education: handbook of theory and research.

[13] Käufer S, Chemero A (2015). Phenomenology: an introduction.

[14] Moustakas CE (1994). Phenomenological research methods.

[15] Lester S (1999). An introduction to phenomenological research [Internet].

[16] Moran D (2000). Introduction to phenomenology.

[17] Patton MQ (2015). Qualitative research & evaluation methods: integrating theory and practice.

[18] Seidman I (2013). Interviewing as qualitative research: a guide for researchers in education and the social sciences.

[19] Glesne C (2011). Becoming qualitative researchers: an introduction.

[20] Saldaña J (2013). The coding manual for qualitative researchers.

[21] Creswell JW (2013). Qualitative inquiry and research design: choosing among five approaches.

[22] Glesne C (2016). Becoming qualitative researchers: an introduction.

[23] Peskin A (1988). In search of subjectivity—one’s own. Educ Res.

[24] Young AP, Hernon P, Powell RR (2006). Attributes of academic library leadership: an exploratory study of some Gen-Xers. J Acad Librariansh.

[25] Altbach PG, Gumport PJ, Berdahl RO (2011). American higher education in the twenty-first century: social, political, and economic challenges.

[26] Lederman D (2010). No letup from Washington. Inside High Educ [Internet].

[27] Bell S (2008). Keeping them enrolled: how academic libraries contribute to student retention. Libr Issues.

[28] Luther J (2008). University investment in the library: what’s the return? a case study at the University of Illinois at Urbana-Champaign.

[29] Mezick EM (2007). Return on investment: libraries and student retention. J Acad Librariansh.

[30] Volentine R, Tenopir C (2013). Value of academic reading and value of the library in academics’ own words. Aslib Proc.

[31] Vance JM, Kirk R, Gardner JG (2012). Measuring the impact of library instruction on freshman success and persistence: a quantitative analysis. Commun Inf Lit.

[32] Hernon P (2010). Shaping the future: advancing the understanding of leadership.

